# Bone Mineral Density in Adolescent Girls with Hypogonadotropic and Hypergonadotropic Hypogonadism

**DOI:** 10.4274/jcrpe.2228

**Published:** 2016-06-06

**Authors:** Mehmet Nuri Özbek, Hüseyin Demirbilek, Rıza Taner Baran, Ahmet Baran

**Affiliations:** 1 Diyarbakır Children State Hospital, Clinic of Pediatric Endocrinology, Diyarbakır, Turkey; 2 Hacettepe University Faculty of Medicine, Department of Pediatric Endocrinology, Ankara, Turkey; 3 Diyarbakır Children State Hospital, Clinic of Radiology, Diyarbakır, Turkey

**Keywords:** hypogonadism, adolescent, follicle-stimulating hormone, osteoporosis

## Abstract

**Objective:**

Deficiency of sex steroids has a negative impact on bone mineral content. In studies conducted on postmenopausal women and animal studies, elevated follicle-stimulating hormone (FSH) levels were found to be correlated with a decrease in bone mineralization and osteoporosis. The aim of the present study was to evaluate bone mineral density (BMD) in adolescent girls with hypogonadotropic and hypergonadotropic hypogonadism and also to investigate the correlation between FSH level and BMD.

**Methods:**

The study group included 33 adolescent girls with hypogonadism (14 with hypogonadotropic hypogonadism and 19 with hypergonadotropic hypogonadism). FSH, luteinizing hormone, estradiol levels, and BMD (using dual energy x-ray absorptiometry) were measured.

**Results:**

There were no statistically significant differences between the chronological age and bone age of the two patient groups, namely, with hypogonadotropic and hypergonadotropic hypogonadism. There was also no significant difference between BMD z-score values obtained from measurements from the spine and the femur neck of patients in the two groups (p-values were 0.841 and 0.281, respectively). In the hypergonadotropic group, a moderately negative correlation was detected between FSH level and BMD z-score measured from the femur neck (ρ=-0.69, p=0.001), whilst no correlation was observed between FSH levels and height adjusted BMD-z scores measured from the spine (ρ=0.17, p=0.493). FSH level was not found to be an independent variable affecting BMD z-score.

**Conclusion:**

BMD z-scores were detected to be similar in adolescent girls with hypogonadotropic and hypergonadotropic hypogonadism, and FSH levels were not found to have a clinically relevant impact on BMD.

**WHAT IS ALREADY KNOWN ON THIS TOPIC?**

Role of estrogen deficiency and elevated follicle-stimulating hormone (FSH) on bone health have been studied in postmenopausal females and in experimental studies. However, there is a lack of studies conducted on bone mineral density (BMD) in adolescent hypogonadal girls.

**WHAT THIS STUDY ADDS?**

The present study evaluates the BMD in girls with hypergonadotropic and hypogonadotropic hypogonadism and assesses the role of FSH in the development of osteopenia/osteoporosis.

## INTRODUCTION

Estrogen has a positive impact on bone mineralization and its deficiency plays a key role in the development of osteoporosis (1). Bone mineral density (BMD) of women with primary ovarian failure and that of adolescents with hypogonadism has been reported to be low as compared to their healthy counterparts (2).

In gonadectomized mice, estrogen replacement has been shown to increase trabecular BMD (3). Furthermore, in mice model including intact controls, hypophysectomized (HX), ovariectomized (OV), and HX+OV mice, ovariectomy was found to be related with profound bone loss, while hypophysectomy blunted this bone loss due to ovariectomy (4). In addition, Sun et al (5) have shown that elevated follicle-stimulating hormone (FSH) levels enhance osteoclastic bone resorption and lead to hypogonadal bone loss. Likewise, FSH was shown to increase bone loss in OV mice by increasing tumor necrosis factor-alpha (TNF-α) and despite having a severe estrogen deficiency, mice deficient in the beta-subunit of FSH (FSH beta) had lower TNF-α and were thereby protected against bone loss (5,6).

A negative correlation has been reported between FSH and BMD in healthy women (7). On the contrary, some studies showed that BMD was correlated with race and body mass index (BMI) rather than FSH and luteinizing hormone (LH) levels (7,8,9).

In children, bone mass increases with age and reaches 90% of the maximum adult bone mass during the adolescent period (10). BMD reaches peak values in the axial skeleton by age 20 in women, while bone mass in the appendicular skeleton reaches its peak values by ages 17-35 years (10). Sex steroids play a substantial role in the increase of BMD and up to 60% of osteoporosis in adult life may be due to the defect in bone mineralization during early adulthood. In female subjects, estrogen deficiency during puberty causes inadequate bone mineralization with increased risk of osteoporosis (11). In studies conducted on postmenopausal women and in animal studies, elevated FSH has been found to be related with a decrease in bone mineralization and development of osteoporosis. However, to the best of our knowledge, the impact of elevated FSH and LH on BMD has not been evaluated in adolescents. The aim of the present study was to evaluate BMD in adolescent girls with hypogonadotropic hypogonadism and hypergonadotropic hypogonadism and to investigate the relationship between FSH level and BMD.

## METHODS

Hypogonadotropic hypogonadism is defined as a low estrogen level with inappropriately normal or low gonadotropin levels (FSH and LH). Hypergonadotropic hypogonadism is defined as inappropriately low estrogen levels in the presence of elevated gonadotropins (FSH and LH) and absence of secondary sexual characteristics. A total of 33 adolescent girls with either hypogonadotropic (n=14) or hypergonadotrophic hypogonadism (n=19) were included in this retrospective study. There was no history of bone fracture in any of the subjects. The group with hypergonadotropic hypogonadism included adolescent females with Turner syndrome (TS) except for one patient with 46,XX gonadal dysgenesis. The cases with hypogonadotropic hypogonadism were being followed with a diagnosis of idiopathic normosmic hypogonadotropic hypogonadism. 

The data were collected from hospital files. Anthropometric measurements (height, weight) were performed using standard methods and devices. Bone age was assessed using the Greulich-Pyle method (12). Serum calcium, phosphorus, alkaline phosphatase, parathormone, 25 hydroxy vitamin D, free thyroxine, thyrotropin, FSH, LH, and estradiol levels were measured.

Patients with concomitant diseases that potentially affect bone mineral content such as Cushing syndrome, severe malnutrition, anorexia nervosa, hyperthyroidism, obesity, disorders in calcium metabolism (e.g. vitamin D deficient rickets), or those receiving medication that have a positive or negative impacts on bone mineralization (growth hormone, estrogen replacement, corticosteroid therapy etc.) were excluded.

BMD was measured using dual energy X-ray absorptiometry (DXA) method (Hologic QDR-4500, USA) from the anteroposterior L1-L4 spine and femur neck. Total bone mineral content in g/cm2 (BMD) and age-adjusted z-scores (BMD z-score) were calculated. Since the hypergonadotropic hypogonadism group included TS patients who were shorter than the hypogonadotropic group, BMD z-scores were adjusted according to height [height adjusted (HA) BMD- z score]. A BMD z-score <-2 standard deviation (SD) was considered as low for age, a low BMD associated with pathological bone fractures was considered as osteoporosis (13,14,15).

### Statistical Analysis

Statistical analysis was carried out using IBM Statistical Package for the Social Sciences (SPSS) 21.0 for Windows statistical software (Armonk, New York, USA). Shapiro-Wilk test was used to test the normality of distribution of the data. Ratios were compared using χ2 test or Fischer’s exact test. Means were compared using independent sample t-test in normally distributed data and medians using Mann-Whitney U test for non-normally distributed data. Spearman rank test was performed for correlations. A multivariate linear regression analysis was performed to test the effect of independent variables on the BMD z-scores. Data were expressed as mean ± SD (range). A p-value <0.05 was considered as statistically significant.

The study was approved by the Institutional Board of Diyarbakır Gazi Yaşargil Training and Research Hospital.

## RESULTS

**Figure 1:**
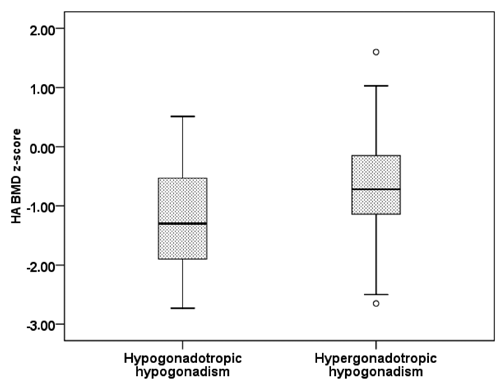
Height adjusted bone mineral density -z scores of patients with hypogonadotropic and hypergonadotropic hypogonadism measured from the spine (L1-L4) were not statistically different. HA BMD: height adjusted bone mineral density

The auxological, biochemical, and hormonal characteristics of the patients are shown in Table 1. There was no statistically significant difference between BMD z-scores of patients with hypogonadotropic hypogonadism and hypergonadotropic hypogonadism (Table 1 and Figure 1). A low BMD z-score (<-2 SD) was detected in 10 of the 14 (71.4%) patients with hypogonadotropic hypogonadism and in 17 of the 19 (89.5%) patients with hypergonadotropic hypogonadism (p=0.363). When the BMD z-score was adjusted for height, the frequency of having a BMD z-score <-2 SD was only 3/14 (21.4%) for the hypogonadotropic group and 3/19 (15.8%) for the hypergonadotropic group (p=0.510).

Spearman rank test revealed that chronologic age moderately negatively correlated with HA BMD z-score in the measurements from the spine, while such a correlation was not found in BMD z-score measurements from femur neck. A weak positive correlation was detected between height standard deviation score (SDS) and BMD z-score measured from femur neck. FSH was weakly negatively correlated with BMD measured from femur neck, whereas a weak positive correlation was found between FSH and HA BMD z-score measured from the spine. No correlations were detected between BMI, estradiol, LH values and BMD z-scores measured from either spine or femur neck (Table 2).

When Spearman rank test was performed in patients with hypogonadotropic hypogonadism, a strong negative correlation was detected between chronologic age and HA BMD z-score measured from the spine, whilst no correlation was detected with BMD z-score measured from femur neck. In the hypergonadotropic group, chronologic age demonstrated a weak negative correlation with HA BMD z-score measured from the spine, whilst no correlation was detected with BMD z-score measured from the femur neck. Besides, in the hypergonadotropic group, BMD measured from the femur neck showed a moderate positive correlation with height SDS and a moderate negative correlation with gonadotropin (FSH, LH) levels (Figure 2A). However, there was no correlation between FSH level and HA BMD z-score measured from the spine (Figure 2B and Table 3). In addition, the correlation analysis performed in patients with hypogonadotropic hypogonadism did not show any significant correlation between FSH level and BMD z-score measured neither from spine nor from femur neck (Figure 2C, 2D).

Multivariate linear regression analysis revealed height SDS as an independent factor affecting BMD z-score measured from spine and femur neck, whereas BMI was found as an independent factor affecting only BMD z-score measured from femur neck (Table 4). FSH was not found as an independent factor affecting the BMD measured neither from spine nor from femur neck.

## DISCUSSION

In the present study, a low age-adjusted BMD z-score (<-2 SD) measured from the spine was detected at a high rate in patients with both hypogonadotropic and hypergonadotropic hypogonadism. HA BMD z-score values were found to be about 6 fold better than the age-adjusted values. In the hypergonadotropic group, FSH levels were negatively correlated with BMD z-scores measured from the femur neck. This relationship did not exist in the hypogonadotropic group. Also, in the hypergonadotropic group, FSH levels were not correlated with BMD measured from the spine and these levels were not found to be an independent factor affecting BMD z-score.

Age and height have previously been reported to be negatively correlated with BMD z-scores. A positive correlation has been reported between weight and BMI and BMD (9,16). Similarly, in our study, chronologic age was negatively correlated with HA BMD z-score measured from the spine whilst not correlated with BMD z-score measured from the femur neck. However, since we have adjusted BMD z-score (spine) according to height, we did not detect a correlation between height SDS and HA BMD z-score. This was consistent with the results of previous pediatric age group studies (13,17,18). The reason that BMD z-score was negatively correlated with chronologic age in our patients with hypo- and hypergonadotropic hypogonadism might be the longer duration of the lack of estrogen, thereby inadequate bone mineralization. On the other hand, prolonged hypogonadism, delayed puberty, and lack of pubertal growth spurt may also have caused the short stature and this may have affected the BMD z-score results (15). Thus, it is recommended that measurements of BMD in children with delayed growth or puberty be adjusted for height or height-age or compared with reference values of age-, gender-, and height-specific z-scores (15). Similarly, height- and age-adjusted BMD z-scores in our cohort were found to be better than only age-adjusted values. Also, the frequency of <-2 SD BMD z-scores was significantly lower when the scores were adjusted by age and height.

It is stated that increase in BMI primarily affects the BMD of the body regions, such as femur neck, which has a higher exposure rate to physical stress compared to the other regions (19). Similarly, in our study, BMI was found to be an independent factor affecting BMD z-scores measured from the femur neck. However, femur neck is not recommended for BMD measurement in children and this relationship between BMI and BMD may not have clinical relevance.

Although not statistically significant, a lower BMD was detected in hypergonadotropic hypogonadism patients, most of whom were cases of TS. This finding was consistent with the results from previous reports indicating an association of TS with increased risk of osteopenia and osteoporosis, while the pathogenesis of this association is still controversial (16,19,20). The impact of low estrogen levels on BMD have been shown in TS patients who demonstrated an improvement in BMD with estrogen replacement and also attained spontaneous puberty (19,20). TS is also characterized with short stature, metaphyseal changes, and vertebral abnormalities that can affect the BMD measurements. The mechanisms through which estrogen deficiency and chromosomal abnormality affect bone mineralization in TS have not yet been clarified (20). By contrast, with due regard to the above-mentioned queries, when BMD z-scores were adjusted for height, our results surprisingly revealed better BMD z-scores in the hypergonadotropic group. This has brought new challenges to our understanding of osteopenia/osteoporosis in TS subjects. Therefore, due to the risk of underestimating osteopenia/osteoporosis in this group of patients, adjusting the BMD z-score according to height in TS patients still remains a controversial issue.

As osteoporosis stands as a major health issue in post-menopausal females who have a typical hormone profile of hypergonadotropic hypogonadism, the relationship between osteoporosis, estrogen, and gonadotropins has been a research subject for many years. Thus, there are studies demonstrating the direct effects of FSH on bone turnover and bone mass in animal models, followed by further studies evaluating this relationship in human female subjects (5,6,7). A negative correlation has been reported between FSH and BMD in large scale studies conducted on adult females most of whom were post-menopausal subjects with hypergonadotropic hypogonadism (7,21,22). While the detection of a negative correlation between FSH level and BMD z-score measured from the femur neck in the subjects of our study was consistent with the results in adult studies, lack of a correlation with HA BMD z-score measured from the spine in our hypergonadotropic group and a lack of correlation with BMD measured from both spine and femur neck in the hypogonadotropic group deviated from these previous studies. The negative relationship between FSH and BMD in the hypergonadotropic group may be due to the more severe estrogen deficiency in cases with higher gonadotropin levels. The reason why estradiol levels were not found to be related to BMD in our cohort was thought to be due to the low estrogen levels in both groups and also to the low sensitivity of estrogen assays. In addition, we did not have age-matched controls to compare the estradiol levels and their impact on BMD z-scores.

Nevertheless, although there was no correlation between FSH and BMD when measured from the spine, a negative correlation was detected between FSH and BMD when the measurements were made from the femur neck. This finding was consistent with the results in adult studies indicating a predominance of FSH effect in physically stressed body regions compared to other regions. However, in children, measurement of BMD from the spine is thought to reflect bone mineral content better than measurements obtained from the femur neck and is a preferred location (15). Therefore, the negative correlations with FSH and BMD derived from measurements from the femur neck may not have any clinical relevance.

In recent studies performed in mice, FSH given as daily injection or infusion did not affect bone mineral content. These results are in accord with findings indicating that FSH has no direct effect on BMD (23,24). In these recent studies, it was shown that FSH has no effect on human mononuclear cell precursors or on the osteoclastogenesis cell line RAW 264.7 (23,24). Similarly, in our study, the negative correlation between FSH and BMD z-score (femur neck) in our hypergonadotropic group and the lack of such correlation in our hypogonadotropic group was attributed to the more severe estrogen deficiency in patients with the higher FSH rather than a direct effect of FSH.

Limitations of this current study was the smallness of the sample and also the lack of an age-matched healthy control group. Our inability to measure the bone mineral markers of osteoclastogenic and osteoblastogenic activities and their relationship with hormonal and DXA measurements was another limitation. We also did not have the means to evaluate the microarchitecture of the bones, an exploration which might have yielded a key on bone quality.

In conclusion, this study which evaluated BMD in adolescent girls with hypogonadotropic and hypergonadotropic hypogonadism revealed a low BMD z-score in the majority of cases in both groups. However, when adjusted for height, a marked improvement was observed, particularly in BMD z-scores of the hypergonadotropic group. FSH level was not found as an independent factor affecting BMD z-score. The negative correlation between FSH and BMD z-score measured from the femur neck was attributed to the more severe estrogen deficiency. There is still a need for larger scale future studies to further elucidate the role of estrogen, gonadotropins, and auxological parameters on bone mineralization in children and adolescents with hypogonadism.

## Ethics

Ethics Committee Approval: Institutional Ethical Comittee of Diyarbakır Gazi Yaşargil Research and Training Hospital, Informed Consent: Obtained from the legal guardians of participants.

Peer-review: External peer-reviewed.

## Figures and Tables

**Table 1:**
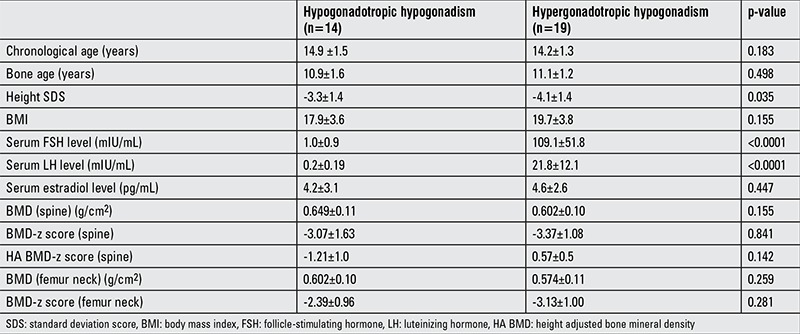
Clinical and laboratory characteristics of patients with hypogonadotropic hypogonadism and hypergonadotropic hypogonadism

**Table 2:**
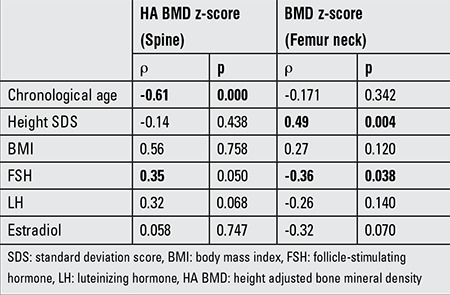
Spearman rank test for chronologic age, height standard deviation score, body mass index, follicle-stimulating hormone, luteinizing hormone, and estradiol levels vs. bone mineral density z-score measured from spine and femur neck

**Table 3:**
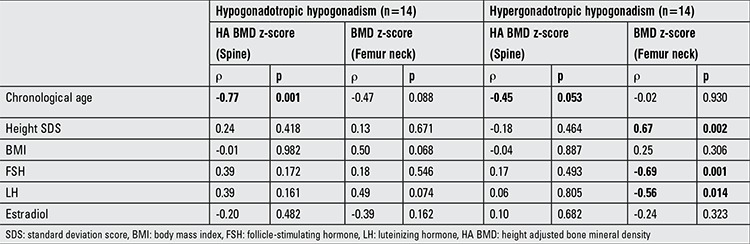
Spearman rank test for chronologic age, height standard deviation score, body mass index, follicle-stimulating hormone, luteinizing hormone, and estradiol vs. bone mineral density z-score/height adjusted bone mineral density z-score measured from spine and femur neck in hypogonadotropic and hypergonadotropic hypogonadism groups

**Table 4:**
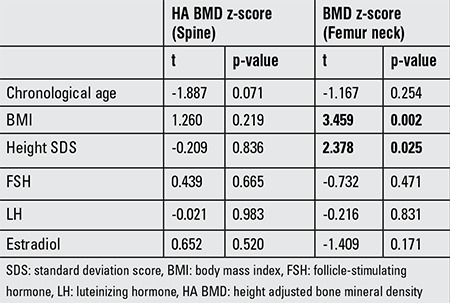
Multivariate linear regression analysis of effect of independent variables on bone mineral density z-scores

**Figure 2 (A, B):**
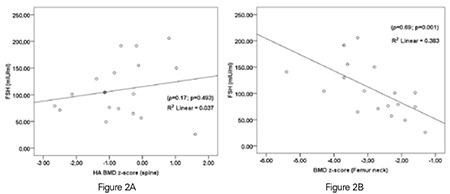
In patients with hypergonadotropic hypogonadism, there was no correlation between follicle-stimulating hormone levels and bone mineral density z-score measured from the spine, whereas a statistically significant negative correlation was detected between follicle-stimulating hormone levels and bone mineral density z-score measured from the femur neck. HA BMD: height adjusted bone mineral density, FSH: follicle-stimulating hormone

**Figure 2 (C, D):**
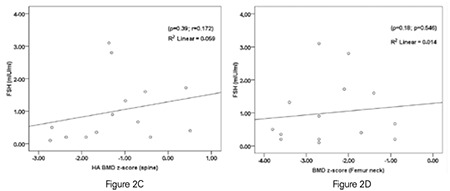
(C, D) In patients with hypogonadotropic hypogonadism, there was no correlation between follicle-stimulating hormone levels and neither height adjusted bone mineral density z-score measured from the spine nor bone mineral density z-score measured from the femur neck. HA BMD: height adjusted bone mineral density, FSH: follicle-stimulating hormone
